# Editorial: Insights in gynecologic surgery 2021

**DOI:** 10.3389/fsurg.2023.1219534

**Published:** 2023-07-10

**Authors:** S. Cianci, S. Gueli Alletti

**Affiliations:** ^1^ Department of Human Pathology of Adult and Childhood “G. Barresi”, Unit of Gynecology and Obstetrics, University of Messina, Messina, Italy; ^2^ Obstetrics and Gynecology Unit, Department of Woman and Child, Ospedale Buccheri La Ferla Fatebenefratelli, Palermo, Italy

**Keywords:** gynecologic surgery, gynecologic oncology, surgical techniques, endoscopic gynecologic surgery, robotic gynecologic surgery

**Editorial on the Research Topic** Insights in gynecologic surgery 2021

Over the last decade, gynecologic surgery has gained significant importance in clinical practice and the scientific panorama.

The technological innovation in robotics, ultra-minimally invasive instruments, and high-performance electrosurgical instruments opened up new frontiers in each surgical specialty, and gynecologic surgery has clearly been involved in this process (1, 2).

Ultra-minimally invasive procedures such as single port, mini-laparoscopy, or percutaneous laparoscopy have accordingly reduced the impact of surgery and the length of hospital stay, in addition to significantly improving the cosmetic outcome (3, 4). This aspect has also been shown to play a role from a psychological point of view, especially in oncologic patients (5). The effect is an improved quality of life, greater adherence to therapies, and better results.

Along with technological development, knowledge of intra- and peri-operative care has improved surgical outcomes, as demonstrated by studies investigating the benefits of fluid and pain-killer balance during care and the importance of physical activity and nutrition in the period before and after surgery (6).

Finally, the importance of pre-operative and intra-operative imaging has led to relevant changes in the surgical approach, especially in gynecologic surgery, allowing for more precise surgery and maintaining adequate standards while reducing invasiveness. For instance, the benefits of visualizing anatomical structures differentiating tissues, solid structures, or vascular perfusion allow us to perform more accurate procedures than in the past (7, 8).

It was an honor and a pleasure for us to serve as Guest Editors of the Research Topic of *Frontiers in Surgery*, specifically its section on *Obstetrics and Gynecologic Surgery* called “Insights in Gynecologic Surgery: 2021”.

We are proud to present a series of articles by renowned specialists in the fields of gynecology and obstetric surgery. All the authors involved in this special issue have made an important contribution to the scientific panorama, which has allowed for improved clinical practice. This issue provides a comprehensive overview of the new developments in gynecologic and obstetric surgery, and the published articles are related to interesting and sometimes rare arguments.

We believe that the topics reported in this special issue will be of interest to a large number of audiences, such as academic researchers, clinicians in gynecology, and obstetrics, surgeons, and even students and trainees in surgical specialties.

The issue begins with the case series by Emery et al. entitled “*Iatrogenic parasitic leiomyoma: The surgeon's invisible hand*”. The authors reported a series of three cases of parasitic leiomyoma after unprotected intra-abdominal morcellation. Considering the patients' characteristics, the authors reported the possibility of pelvic inflammation as a risk factor for parasitic leiomyoma development.

The second work, by Liu et al. is entitled “*Transvaginal single-port extraperitoneal laparoscopic sacrospinous ligament fixation for apical prolapse: A single-center case series*”. The authors published interesting data on a series of nine patients presenting with apical pelvic organ prolapse and treated using a single-port extraperitoneal approach with the aim of evaluating the safety and efficacy of this surgical approach.

The third article by Zhao et al. entitled “*Strengthen the sacral ligament and paravagina by equilibrium control severe pelvic organ prolapse*” reported on a series of 76 patients who underwent a modified surgical technique for the treatment of pelvic organ prolapse consisting of a combination of sacrocolpopexy and sacral ligament conjunction.

The fourth contribution, by Lv et al. entitled “*Study of the effect of pain on postoperative rehabilitation of patients with uterine malignant tumor*”, was focused on evaluating the relationship between postoperative pain and quality of life in gynecologic oncology patients. The authors analyzed data from 102 patients to evaluate the influence of surgical approach and postoperative rehabilitation.

Hao et al. presented the fifth article, entitled “*The impact of omentectomy on cause-specific survival of stage I-IIIA epithelial ovarian cancer: a PSM-IPTW analysis based on SEER database*”. They conducted research using the SEER database to evaluate the usefulness of omentectomy at the time of surgical treatment for ovarian cancer patients. Based on the results, the authors reported that omentectomy in the case of non-macroscopic disease is not associated with survival benefits.

The sixth study, by Gulino et al. entitled “*Isolated tubal torsion in a term pregnancy: case report and systematic review of literature of the last ten years*” reported a rare case of tubal torsion in a pregnant patient and the possible management based on patient characteristics and gestational age.

The final article in this special issue, by Sahin et al. is “*Are Cesarean section and appendectomy in pregnancy and puerperium interrelated? A cohort study*”. This work was focused on evaluating the possible relationship between the cesarian section and appendectomy. A total of 11,513 patients were enrolled in the study, and the authors suggested that the acute abdomen in the post-partum period could be more often related to acute appendicitis, especially in women who underwent cesarian sections.

We would like to thank all the authors for their commitment to publishing these interesting articles. which may constitute a possible resource for clinicians and researchers.
